# Evolving Therapeutic Strategies to Exploit Chromosome Instability in Cancer

**DOI:** 10.3390/cancers9110151

**Published:** 2017-11-01

**Authors:** Laura L. Thompson, Lucile M-P. Jeusset, Chloe C. Lepage, Kirk J. McManus

**Affiliations:** 1 Department of Biochemistry & Medical Genetics, University of Manitoba, Winnipeg, MB R3T 2N2, Canada; laura.thompson@umanitoba.ca (L.L.T.); jeussetl@myumanitoba.ca (L.M-P.J.); lepagec@myumanitoba.ca (C.C.L.); 2 Research Institute in Oncology and Hematology, CancerCare Manitoba, Winnipeg, MB R3E 0V9, Canada

**Keywords:** chromosome instability, cancer, intratumoral heterogeneity, precision medicine, synthetic lethality, combinatorial chemotherapy

## Abstract

Cancer is a devastating disease that claims over 8 million lives each year. Understanding the molecular etiology of the disease is critical to identify and develop new therapeutic strategies and targets. Chromosome instability (CIN) is an abnormal phenotype, characterized by progressive numerical and/or structural chromosomal changes, which is observed in virtually all cancer types. CIN generates intratumoral heterogeneity, drives cancer development, and promotes metastatic progression, and thus, it is associated with highly aggressive, drug-resistant tumors and poor patient prognosis. As CIN is observed in both primary and metastatic lesions, innovative strategies that exploit CIN may offer therapeutic benefits and better outcomes for cancer patients. Unfortunately, exploiting CIN remains a significant challenge, as the aberrant mechanisms driving CIN and their causative roles in cancer have yet to be fully elucidated. The development and utilization of CIN-exploiting therapies is further complicated by the associated risks for off-target effects and secondary cancers. Accordingly, this review will assess the strengths and limitations of current CIN-exploiting therapies, and discuss emerging strategies designed to overcome these challenges to improve outcomes and survival for patients diagnosed with cancer.

## 1. Introduction

Cancer is a significant global concern with more than 14 million new diagnoses and 8 million deaths attributed to this disease each year [1]. Despite the significant advances made in screening, detection [2], and treatment of early-stage cancers [3], many patients still present with late stage disease at diagnosis. For example, colorectal cancer is the second leading cause of cancer-related deaths in the United States, and despite increased screening efforts, 21% of patients are initially diagnosed with metastases [4], while ~50% will inevitably develop metastatic disease [5]. As metastatic cancers are generally associated with poorer prognoses and outcomes (e.g., 5-year survival of 13% for metastatic colorectal cancer [6]), improved treatment strategies designed to better combat both primary and metastatic disease are in dire need.

Chromosome instability (CIN) is an enabling feature of cancer [7] that is defined as an increase in the rate at which whole chromosomes (numerical CIN) or chromosomal fragments (structural CIN) are gained or lost, typically resulting in aneuploidy or abnormal DNA content [8,9]. CIN is frequently observed in the majority of cancer types, including both hematologic and solid tumors, but is arguably best understood in colorectal cancer, where it is observed in up to 85% of all cases [8,10]. The numerical and/or structural chromosome alterations associated with CIN often occur as a result of defects in numerous biological pathways, including centrosome dynamics, mitotic spindle assembly, kinetochore-microtubule attachment, sister chromatid cohesion, replication stress, and DNA damage repair [11,12,13,14,15,16,17,18,19,20,21,22]. Numerical CIN underlies aneuploidy, and leads to gene copy number alterations for contiguous gene sets, while structural CIN can induce specific gene amplifications, deletions, or translocations [9]. Despite distinct definitions, numerical and structural CIN are intricately linked, frequently occurring within the same tumor cells and often exhibiting a causal relationship [9,23]. For example, DNA double-stand breakages (structural CIN) can occur as a result of forces acting on missegregated chromosomes (numerical CIN) that remain “trapped” within the cleavage furrow during cytokinesis [23]. Overall, CIN promotes oncogenesis by increasing the rate at which key genes—including oncogenes, tumor suppressor genes, DNA repair genes, and apoptotic genes—are gained, lost, or altered [24]. Accordingly, CIN drives cellular transformation [25,26], cancer progression [27], intratumoral heterogeneity (ITH) [28], multi-drug resistance [29,30], tumor recurrence [29,30], and poor patient outcomes [29,31].

Conceptually, a precision medicine strategy that selectively exploits the aberrant genes (CIN genes) or pathways causing CIN would be effective in a myriad of cancers (at both primary and metastatic sites), and would reduce and/or eliminate many of the off-target side effects associated with many current chemotherapeutics [28,31,32,33]. Recent technological advancements in the detection of CIN, coupled with novel therapeutic strategies designed to exploit the molecular origins of CIN, are beginning to emerge. In fact, chemotherapeutics that exploit CIN have already shown promise in the clinic, both as single agents and as combination therapies (detailed below), and support CIN as a strong therapeutic target. In this review, we will present the relationship between CIN and cancer, discuss current and emerging therapeutic strategies that exploit CIN, and focus on the concerns and potential solutions for their effective clinical use. 

## 2. Assessing CIN In Vitro and in Patient Tumor Samples

To successfully develop and employ CIN-exploiting therapies, CIN must first be accurately defined, identified, and assessed within a given tumor sample. In this respect, an important distinction must be drawn between CIN and genome instability, as different underlying biological mechanisms may necessitate the use of distinct treatment approaches. Genome instability is an abnormal state characterized by a high prevalence of genomic alterations, and can include mutations in nucleic acid sequence, aberrant chromatin modifications, chromosomal rearrangements, or aneuploidy. Although many aberrant pathways give rise to genome instability, CIN is the predominant form of genome instability, and specifically refers to an increase in the rate of chromosome or chromosome fragment gains or losses. Accurately assessing CIN mandates the use of approaches capable of characterizing the rate of chromosomal changes over time, or alternatively, single-cell approaches capable of assessing chromosomal heterogeneity within a given population [34]. As direct measurement approaches are often labor intensive, additional indirect techniques that evaluate indicators suggestive of CIN are also frequently employed.

Given that CIN is frequently associated with aneuploidy, indirect techniques, such as gene expression signatures and comparative genomic hybridization (CGH) designed to evaluate aneuploidy, are often used as a proxy for CIN (Table 1). For example, the CIN70 gene signature was developed to assess the expression of 70 genes whose overexpression correlates with aneuploidy. More specifically, high CIN70 scores are associated with poor prognosis in multiple cancer types, including breast and lung cancers, and were increased in metastases [31]. Similarly, CGH was employed to compare the frequency of chromosomal imbalances in primary colorectal tumors and brain metastases, revealing a higher degree of segmental aneuploidy in metastatic lesions [35]. Collectively, these studies show that aneuploidy, which often arises from CIN, favors cancer progression and metastatic dissemination. However, since aneuploidy can develop by mechanisms independent of CIN and remain stable over time [36], the presence of aneuploidy does not necessarily signify CIN. Furthermore, since the above methods employ pooled DNA samples isolated from large numbers of cells (10^3^–10^6^ cells), they are incapable of measuring the level of cell-to-cell heterogeneity in chromosome numbers and structures that are characteristic of CIN. 

To circumvent the population-based averaging effects of standard approaches, many groups have pioneered quantitative, single-cell approaches to assess multiple CIN phenotypes, and are now applying them in various cancer contexts. Currently, only single-cell approaches, such as DNA image cytometry and fluorescence in situ hybridization (FISH), are capable of evaluating and assessing CIN. As these methods are time consuming and costly, surrogate markers, such as changes in nuclear areas and micronucleus formation (Table 1), can offer an initial alternative approach to rapidly evaluate samples [38]. If the preliminary results are suggestive of CIN (i.e., significant changes in nuclear area heterogeneity or micronucleus formation), confirmation with an additional single-cell method can be performed. Flow cytometry and DNA image cytometry (Table 1) can be employed to measure the DNA content of individual cells from patient samples, including formalin-fixed and paraffin-embedded (FFPE) tissues. Further, cell-to-cell heterogeneity in DNA content can be assessed through the Stemline Scatter Index (SSI, Table 1), and high SSI scores are indicative of CIN-positive tumors [40,41]. However, this method is incapable of distinguishing numerical from structural CIN, which may provide mechanistic insights and inform the drug selection process. 

In general, interphase FISH is the most commonly employed method to assess both structural and numerical CIN in patient samples (Table 1) [8,34,44,45,46,47]. Major benefits of this approach are that it assesses cell-to-cell heterogeneity and can also evaluate CIN in sequential samples isolated from patients, to monitor disease progression and treatment response. In 2017, Penner-Goeke et al. [42] employed interphase FISH and assessed CIN in serial samples collected from the ascites of women with ovarian cancer. They showed that CIN is both present and dynamic in serial samples, and increases in women with resistant disease. However, this method can only evaluate changes in small subsets of chromosomes or chromosomal regions at a time. Thus, more exhaustive approaches, like single-cell CGH or single-cell sequencing, two techniques currently under development, may ultimately replace interphase FISH. Single-cell CGH and single-cell sequencing (Table 1) are ideally suited to assessing CIN, as they combine accurate assessment of cell-to-cell heterogeneity with an in-depth analysis of the chromosomal changes and genetic alterations contained within individual cells of a given tumor. However, significant cost reductions coupled with improvements in multiplexing capacity and clinical bioinformatics are required before these tools become integrated within the clinic. 

Exploiting CIN for therapeutic purposes necessitates not only accurate detection and assessment of CIN, but also requires a fundamental understanding of the underlying aberrant molecular pathways driving CIN and oncogenesis. The CIN phenotype is complex, derived from alterations in a diverse array of genes and cellular pathways, and in many instances, the causative link to cancer has yet to be fully understood. Much of our current knowledge of CIN originates from studies in model organisms, including budding yeast, that predict there may be at least 2300 CIN genes contained within the human genome [39,48]; however, only a small proportion of human CIN genes have been identified to date. The majority of known human CIN genes function in readily apparent pathways, including DNA damage repair and chromosome dynamics. Nevertheless, cross-species approaches suggest that additional and less intuitive pathways, including lipid metabolism, proteasomal degradation, and tRNA synthesis may also exhibit important roles in CIN and oncogenesis [48]. Thus, a concerted effort is needed to identify and delineate the aberrant molecular mechanisms that underlie this complex phenotype.

## 3. Current and Emerging CIN-Exploiting Therapies

**Figure 1:**
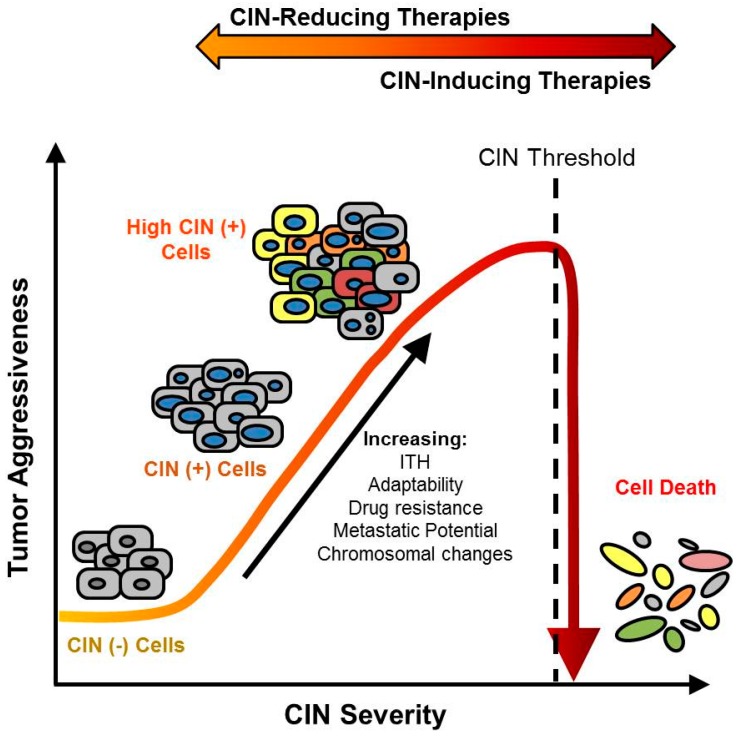
Therapeutic Strategies to Exploit Chromosome Instability (CIN) in Cancer. Schematic presenting the relationship between increasing CIN and key tumor features (e.g., adaptability, intratumoral heterogeneity (ITH), drug resistance, metastatic potential and chromosomal changes). Two alternative therapeutic strategies that exploit CIN are presented (arrow, top). (1) CIN-reducing treatment strategies suppress CIN in CIN+ tumors to slow and/or prevent acquisition of additional chromosomal alterations. Ideally, this will hinder tumor adaptability, cancer cell evolution, and the acquisition of drug resistance, thereby reducing tumor aggressiveness; (2) Alternatively, CIN-inducing strategies seek to generate extensive levels of numerical and/or structural CIN beyond a critical threshold (black dotted line) to induce cell death. +: positive; −: negative.

Currently, there are two fundamental strategies for exploiting CIN in cancer, that are generally referred to as either CIN-reducing or CIN-inducing (Figure 1) [11,12,13]. Conceptually, CIN-reducing approaches aim to slow the rate of CIN, and typically function by inhibiting the abnormal processes leading to chromosome missegregation or structural changes in CIN-positive cancer cells. In doing so, this strategy seeks to prevent the acquisition of further chromosomal alterations, to minimize ITH and tumor adaptability, with the goals of limiting cancer progression and drug resistance. While several in vitro studies have successfully employed chemical or genetic CIN-reducing approaches and identified promising targets [49,50,51,52,53], many have yet to be translated into the clinic. Conversely, CIN-inducing therapies aim to exacerbate CIN, generating extreme levels of chromosome missegregation and/or DNA damage to induce cell death [11,54,55,56]. Support for this approach is garnered from the paradoxical relationship observed between extensive CIN (as measured by gene expression signature CIN70 and FISH probe enumeration) and improved patient outcome in various cancers, including breast, ovarian, gastric, and lung [37,57]. Interestingly, within these studies, the poorest patient outcomes were associated with intermediate levels of CIN, rather than extreme levels [37]. These observations suggest a threshold of CIN may exist that, when exceeded, induces cell cytotoxicity (Figure 1). Unfortunately, these theoretical CIN thresholds may be cell type- or cancer-specific, or may vary with the genetic context of the cell. For instance, extreme CIN (as measured by CIN70, CGH, and centromeric FISH probes) was found to correlate with improved long-term survival in estrogen receptor (ER)-negative breast cancer, but poorer outcome in ER-positive breast cancer patients [58]. These observations highlight the complexity associated with selecting an appropriate CIN-exploiting therapy for different cancer types, as CIN thresholds that are compatible with either viability or death are not well established. 

Many CIN-reducing and -inducing therapies target biological processes commonly associated with numerical CIN, like mitosis and chromosome dynamics (Table 2). For example, several drugs bind to tubulin subunits of the mitotic spindle apparatus to disrupt normal chromosome segregation. One such drug, Paclitaxel, is a taxane commonly employed in breast, ovarian, and lung cancers, that binds to β-tubulin. Paclitaxel binding stabilizes microtubule polymers to prevent their assembly or disassembly, which blocks normal chromosome congression to the metaphase plate, resulting in a mitotic (prometaphase-like) arrest. At clinically relevant doses, Paclitaxel induces CIN by generating multi-polar spindles that adversely impact chromosome segregation, and ultimately leads to cell death [54]. Additional taxanes and other tubulin-binding drugs (e.g., epothilones and vinca alkaloids (Table 2)) are also employed clinically, and typically function by altering the rates of microtubule polymerization/depolymerization, to interfere with spindle dynamics and chromosome segregation [59].

The spindle assembly checkpoint (SAC) is another common therapeutic target in many cancer types that has important implications for CIN (Table 2). In general, the SAC monitors microtubule attachment to kinetochores, ensuring that appropriate bi-orientation is achieved. Bi-orientation refers to a process whereby microtubules emanating from opposite spindle poles are captured by the kinetochores of sister chromatids. The SAC is a negative regulator of the anaphase promoting complex/cyclosome (APC/C) that restricts anaphase entry until all kinetochores have achieved proper bi-orientation [94]. Several pharmacological studies have shown that APC/C inhibition with various compounds induces a prolonged mitotic arrest that is associated with reduction in chromosome segregation errors, and a decrease in CIN [66,68,69]. Conversely, SAC inhibitors have been developed (Table 2) that enhance APC/C activation, and promote premature exit from mitosis that is associated with increases in chromosome segregation errors and CIN [62,63]. 

More recently, the Aurora family kinases have garnered considerable attention as CIN-inducing therapies (Table 2). Members of this kinase family also exhibit critical roles in mitotic chromosome segregation, and are important regulators of centrosome biology, mitotic spindle assembly, and microtubule–kinetochore attachments. AZD1152 [56,95] is an Aurora Kinase B inhibitor that promotes endoreduplication (replication of DNA in the absence of cell division [96]), the formation of large multinucleated cells, and apoptosis [95]. AZD1152 inhibits growth of human colon, lung, and hematologic tumor xenografts in immunodeficient mice [97], and is now undergoing clinical trials in both solid and hematological cancers [95,98].

Mitotic microtubule motor proteins also represent promising CIN-exploiting therapeutic targets in the clinic (Table 2). Kinesins, for example, exhibit fundamental roles in mitotic spindle assembly and establishment of bipolar spindle attachments [99,100]. Inhibitors like Ispinesib [101] prevent spindle assembly and proper bi-orientation, and induce prolonged SAC-induced arrest (mitotic arrest) that typically underlies apoptosis. In some instances, cells may escape the SAC, resulting in aberrant chromosome segregation and CIN [19]. However, in CIN-positive cancer cells that harbor supernumerary centrosomes (more than 2), inhibiting kinesin motor proteins may restore normal bipolar spindle formation through centrosome clustering, to effectively reduce CIN [102]. Therefore, the pre-existing level of CIN and underlying mechanism(s) responsible for CIN may dictate whether the effect of a particular therapy will be CIN-reducing or CIN-inducing. Interestingly, many cancer cells demonstrate adaptation to supernumerary centrosomes through an analogous centrosomal clustering mechanism, suggesting that maintaining a bipolar spindle may favor cancer cell viability [103,104]. In these cells, therapies that inhibit, rather than induce centrosomal clustering, may prove therapeutically beneficial. In fact, in vitro studies in breast cancer cell lines have shown that treatment with the centrosome declustering compound 5-nitro-N-(3-pyridinylmethyl)-2-furancarboxamide (CCCI-01), induces multipolar spindle formation, CIN, and apoptosis [88]. CCCI-01 differs from more general mitosis-targeting drugs like Paclitaxel, as its effects are restricted to cells with supernumerary centrosomes. Thus, treatment with CCCI-01 may help improve cancer specificity and reduce off-target toxicities associated with more general chemotherapies, like Paclitaxel. 

Beyond the numerical CIN-targeted therapies highlighted above, additional precision medicine strategies that induce structural CIN have also begun to show clinical utility in several cancer types. For example, poly(ADP-ribose) polymerase (PARP) inhibitors, like Olaparib, exert their therapeutic effects through a genetic strategy referred to as synthetic lethality (detailed below). PARP functions in numerous DNA repair pathways, and in particular, the base excision repair pathway that is required for the accurate detection and repair of DNA single-strand breaks [105]. In the presence of PARP inhibitors, DNA single-strand breaks persist, and are converted into double-strand breaks during DNA replication in S-phase, due to replication fork collapse [47]. Importantly, many cancers harbor defects in double-strand break repair pathway genes, like *BRCA1* and *BRCA2*, that encode functions within the homologous recombination repair (HRR) pathway [106]. *BRCA1/2*-defective cancer cells exhibit enhanced sensitivity to PARP inhibitors, as they are unable to efficiently repair the resultant DNA double-strand breaks, which leads to cell death [107,108]. Olaparib is currently clinically employed in ovarian cancers harboring *BRCA1/2* deficiencies [109], and has also begun to show promise in *BRCA1/2*-deficient chemoresistant, metastatic breast and prostate cancers [110,111]. Thus, the above information supports the development and use of therapeutics that target numerical and/or structural CIN in cancer, many of which are at early stages of the drug discovery pipeline.

## 4. CIN Increases ITH and Drives Multidrug Resistance

The development and use of single CIN-targeted agents in cancer is associated with specific challenges that must be overcome in order to achieve optimal therapeutic efficacy. Targeting CIN-positive tumors and exploiting CIN for therapeutic gain is primarily impeded by the dynamic and heterogeneous nature of the CIN phenotype. CIN-positive tumors often harbor extensive chromosomal ITH, and acquire chromosomal changes at an increased rate [112]. Therefore, cells harboring alterations that promote metastasis, cell survival and drug resistance (e.g., inhibit drug uptake, drive drug efflux, inactivate/metabolize the drug, or alter cell signaling to mitigate the effects of the drug [112]) are more likely to be present within a CIN-positive tumor, and likely account for the correlation between CIN and highly aggressive, multidrug-resistant cancers [29]. Interestingly, a recent clinical trial investigating Paclitaxel in CIN-positive ovarian cancers revealed intrinsic resistance to taxanes. This “inherent” resistance likely stems from the fact that the same molecular mechanisms targeted by Paclitaxel were already misregulated in those tumors [41]. Thus, it is possible that cancer cells that have genetically adapted to CIN may exhibit intrinsic resistance to drugs that seek to increase CIN through a similar mechanism. 

Gerlinger et al. [113] reports that metastasis, recolonization, and proliferation of CIN-positive tumors often creates metastases with regional ITH that are genetically divergent from both the primary tumor and other metastases. While CIN represents a common target that may be exploited for the treatment of highly heterogeneous tumors and distinct metastatic lesions, extensive ITH may also complicate the process of selecting an appropriate treatment. Genetically heterogeneous tumors may harbor sub-clonal populations exhibiting different rates of CIN, or different underlying mechanisms that drive CIN, resulting in cell-to-cell variation in treatment response. For example, a CIN-inducing drug may increase the level of CIN beyond a critical threshold, and induce death for a subset of tumor cells already exhibiting a “high” level of CIN, whereas the viability of cells initially exhibiting a lower level of CIN may not be compromised. Additionally, administering a centrosome declustering drug would only be appropriate for patients whose cancer cells exhibit supernumerary centrosomes. These examples emphasize the importance of determining tumor CIN status and characterizing the aberrant molecular mechanism(s) underlying the CIN phenotype, to direct the most effective therapeutic strategy for a given cancer patient. 

Extensive CIN and ITH also increase the risk for acquired drug resistance, or the development of resistance to a previously effective treatment [112]. For example, while a single agent treatment approach may kill the majority of cells within a given tumor, a drug-resistant sub-population may persist and expand, resulting in tumor recurrence [114,115]. Indeed, resistance to PARP inhibitors that are initially effective in HRR-deficient cells can occur by re-establishing the reading frame of *BRCA1/2* genes (harboring frame-shift mutations) to restore HRR function [116]. Although single agent targeted therapies do improve overall patient survival, clinical responses can be short-lived, as tumors rapidly evolve to become drug resistant within a few months [114]. For example, resistance to Vemurafenib (a BRAF (B-Raf Proto-Oncogene, Serine/Threonine Kinase) kinase inhibitor) frequently arises in melanoma by oncogenic re-activation of a downstream mitogen activated protein kinase (MAPK) signaling pathway member [117]. Therefore, employing alternative CIN-targeting therapeutic strategies, including the combinatorial treatment approaches discussed below, will be important for the effective treatment of CIN-positive tumors. 

## 5. Potential Risks Associated with CIN-Exploiting Therapies

In addition to the complications associated with drug resistance detailed above, a significant concern with exploiting CIN is the potential risk for off-target effects and the development of secondary cancers. As many CIN-targeted therapies actually promote CIN themselves, the possibility exists that these treatment strategies may induce CIN in non-cancerous cells, and drive the development of de novo (secondary) cancers. Further, if a given CIN-exploiting compound fails to eradicate all cells within a given tumor, the increased rate of CIN may inadvertently create a more aggressive tumor with an enhanced potential to become drug resistant. In this regard, a recent study [19] showed that silencing or inhibition (Monastrol) of KIF11 (Kinesin Family Member 11), a microtubule motor protein required for spindle pole dynamics during mitosis, initially induced monopolar formation and a prometaphase-like arrest; however, these anti-proliferative effects were only transient in both cancerous and immortalized cell lines [19]. Presumably, the cells that escaped the siRNA or Monastrol induced arrest did so through checkpoint (SAC) adaptation or mitotic slippage, and re-entered the cell cycle without undergoing chromosome segregation or cytokinesis. This possibility is supported by their observations of significant increases in nuclear areas and chromosome numbers relative to controls. Accordingly, Monastrol (and perhaps additional KIF11 inhibitors) enhances CIN in both cancerous and immortalized cell lines, which if translated to humans, may promote the development of drug resistant disease, or the induction of secondary tumors. Thus, the experimental findings detailed above may account for the limited benefits observed in initial clinical trials investigating KIF11 inhibitors, like Ispinesib [19].

## 6. Characterizing ITH to Identify Optimal Targets for CIN-Exploiting Therapies

The presence of CIN in tumor cells is synonymous with the development of ITH. As such, ITH challenges the efficacy of CIN-exploiting therapies by promoting intrinsic and acquired drug resistance, which limits the probability of identifying a single effective treatment agent. Overcoming these challenges and identifying optimal targets to exploit CIN will be enabled through a detailed description of the most frequent underlying genetic events driving CIN within a tumor. Unfortunately, such a description is not routinely available within the clinic, although recent technological advancements suggest it may be possible in the future. In particular, deep sequencing of multiple tumor regions/sites can identify common, actionable genetic alterations (see below), while ultra-deep sequencing can identify low frequency (1% or less) variants with important clinical implications, particularly for drug resistance. For example, ultra-deep sequencing of a breast cancer sample identified sub-clones with mutations conferring Lapatinib (HER2 (Human Epidermal Growth Factor Receptor 2) inhibitor) resistance, indicating that an alternative treatment (Trastuzumab) may be more appropriate to reduce the risk of drug resistance and disease recurrence [118]. In addition, sub-clones were identified with actionable mutations in multiple patients that were not identified with less sensitive methods. Thus, as these approaches are integrated within routine clinical practice, they will be instrumental in directing clinical management of the disease.

As CIN drives ITH and distinct sub-clonal populations are likely to be spatially segregated within a tumor, (ultra-)deep sequencing and multi-region sampling are critical to identify common actionable targets [113,119,120]. In renal cell carcinoma, a cancer type that frequently exhibits CIN, studies show that accurate characterization of ITH is crucial to develop optimal therapeutic strategies. Using multi-region sequencing and a tree-based analogy, researchers were able to distinguish ubiquitous “truncal” alterations that occur early in tumor development and are shared by most cells within primary and metastatic lesions, from sub-clonal “branch” alterations that arise later during tumor progression and are only found in a subset of cells [113]. Accordingly, it is the frequent truncal alterations that represent optimal therapeutic targets, as they are common to most tumor cells. Thus, identifying the actionable truncal alterations will be important to deliver highly effective cancer treatments. In this regard, a recent study determined that ~70% of branch alterations in renal cell carcinoma were erroneously identified as truncal mutations, following sequencing of only a single region [119], whereas multi-region sampling and sequencing determined they were sub-clonal, branch events. Thus, multi-sample deep sequencing will be essential to identify the actionable truncal targets, but will also be critical to identify sub-clonal alterations that confer drug resistance.

The identification and targeting of common actionable alterations does not preclude the emergence of drug-resistant populations, particularly in tumors with CIN. For example, strategies targeting gain-of-function alterations associated with increases in gene copy numbers will be rendered ineffective through subsequent loss of whole chromosomes or chromosome fragments involving those specific gene loci. In contrast, loss-of-function alterations stemming from homozygous deletions of CIN genes are unable to be reversed, and are ideal targets to exploit using a synthetic lethal paradigm (detailed below). For example, a multi-region sequencing analysis identified inactivation of Polybromo 1 (*PBRM1*), a component of the SWI/SNF (SWItch/Sucrose Non-Fermentable) chromatin remodeling complex, as a truncal event in several renal cell carcinoma cases [119]. Inactivation of PBRM1 and additional members of the SWI/SNF complexes are associated with CIN [121,122], and a pre-clinical study showed that truncal *PBRM1* alterations may be exploited through CIN-inducing therapies [123].

An additional benefit of characterizing ITH is the ability to identify instances of convergent evolution, which may reveal common drug susceptibilities/targets. In this context, convergent evolution occurs when alterations in distinct genes adversely impact the same CIN pathway (e.g., microtubule dynamics, SAC function or centrosome biology), but within different sub-clonal populations. For example, in renal carcinomas where *PBRM1* inactivation was not deemed a truncal event, many tumor cells still harbored defects in additional SWI/SNF member genes [119]. Therefore, it is predicted that disruption of SWI/SNF function, through defects in a diverse array of genes, will induce CIN through a convergent mechanism, and render each sub-population sensitive to any therapy that exploits SWI/SNF defects. Beyond therapeutic targeting, such instances of convergent evolution also suggest that tumor cells may become dependent on a given CIN pathway for survival and proliferation. Hence, therapies that exploit these CIN pathways are more likely to be effective, and have a reduced potential for the development of drug resistance. Overall, a detailed characterization of the actionable truncal and branch mutations throughout a tumor will be critical, to devise the most effective precision medicine strategy for a given patient.

## 7. Harnessing Synthetic Lethality to Develop Effective and Specific CIN Therapies

Synthetic lethality is defined as a rare and lethal combination of two independently viable gene mutations or deletions [124,125,126], and is an emerging strategy that can be employed to selectively exploit the underlying truncal (and branch) alterations that cause CIN [124,127]. In a cancer context, defects in a CIN gene may be exploited by downregulating or inhibiting a synthetic lethal (SL) interactor (i.e., drug target) to induce tumor-specific killing. Unlike conventional therapies that frequently inhibit gain-of-function alterations associated with oncogenes, synthetic lethality can also exploit loss-of-function alterations associated with the loss of genes like tumor suppressor genes, DNA repair genes, and anti-apoptotic genes. As such, synthetic lethality is ideally suited to exploit the truncal loss-of-function alterations leading to CIN, and is best exemplified by the synthetic lethal targeting of *BRCA1/2*-deficient ovarian cancers with the PARP inhibitor, Olaparib (detailed above) [109]. Furthermore, because truncal alterations are frequently conserved in both primary and metastatic lesions, SL strategies are predicted to be effective in both primary and metastatic disease. Finally, SL strategies are also expected to reduce adverse side-effects and the occurrence of secondary cancers, as they are inherently restricted to targeting cancer cells with defects in CIN genes. Although promising, this aspect remains to be confirmed clinically as the use of SL strategies in patients is still in its infancy.

Beyond exploiting truncal alterations in CIN genes, SL approaches are also predicted to be an effective strategy capable of targeting tumor cells exhibiting convergent evolution. Indeed, genes encoding functions within the same biological pathway often share SL interactors [128,129,130], and thus conceptually, therapies that exploit one defective complex/pathway member are likely to be effective against additional defective members of the same complex/pathway. For example, PARP inhibitors that target *BRCA1*/*2* defects underlying HRR-deficiencies are also effective at killing cancer cells harboring defects in additional HRR genes, like *RAD51*, *ATM*, *ATR*, *CHEK1*, and *RAD54B* [106,131,132,133,134,135,136,137]. These observations suggest that PARP inhibitors like Olaparib, may have clinical utility beyond the prototypic *BRCA1/2*-defective ovarian cancer context. The ability to exploit a broad array of genetic alterations through a single SL treatment (e.g., PARP inhibitor) represents a significant advantage over traditional targeted therapies that are restricted to a single molecular target. In this regard, a number of PARP inhibitors are currently under investigation in clinical trials aimed at assessing their utility in additional HRR-defective contexts and cancer types [60]. In addition, multiple new SL interactions were recently identified in pre-clinical studies involving various CIN genes altered in cancers, such as *RAD54B*, *CHEK2*, *BLM*, *PTEN,* and *TDP1* [128,129,138,139,140]. The SL interactors of these CIN genes are potential therapeutic targets of high interest for future CIN-exploiting therapies.

## 8. Combinatorial Chemotherapeutic Strategies May Circumvent ITH and Drug Resistance Stemming from CIN

ITH poses a significant challenge that must be surmounted to deliver effective cancer treatments. Drug resistance is a frequent outcome of many targeted therapies, and is of particular concern in cancers with CIN. To combat these issues, combinatorial drug treatment strategies that target multiple aberrant pathways may prove more effective than a single agent targeting a single pathway, as there is a reduced probability that a given tumor cell will exhibit pre-existing resistance to multiple drugs [114,141]. Theoretical support for combinatorial treatments comes from mathematical models simulating tumor growth, metastasis, and resistance in response to various treatment strategies [115]. Many recent studies have focused on delineating the genomic changes occurring in response to targeted cancer treatments [114,115,142]. For example, Bozic et al. [114] employed sequencing data generated from multiple spatially and temporally disparate tumor samples, to reconstruct an evolutionary tree of the complex genomic changes that arose during tumor development, progression, and metastasis. Their “Treeomics” approach combined spatial growth patterns and temporal genomic changes to identify the ancestral sub-clones responsible for distinct metastases. Solid tumors typically harbored between 30 and 70 clonal nonsynonymous mutations, with most representing passenger mutations, while 5–10% were driver mutations that provided a selective growth advantage and contributed to oncogenesis [143]. This study also determined that the driver mutations were typically present in 30–100% of primary tumor cells, as well as in the metastatic lesions, and thus identified promising therapeutic targets [115]. Bozic et al. [114] also modelled tumor response to single and combinatorial treatments in pancreatic, colorectal, and melanoma cancer patients with metastatic disease [114]. While single agent therapies were inevitably associated with drug resistance, dual agent strategies resulted in long-term remission for most patients in these simulations (provided no single mutations were present that conferred resistance to both drugs). They further showed that simultaneous treatments, rather than sequential, were most efficacious, and that triple agent strategies were required for large tumors. Thus, Bozic and colleagues [114] developed mathematical models to describe the evolutionary dynamics that arise in response to targeted therapies, and simultaneously uncovered the most frequently altered genes, which may represent ideal therapeutic targets. In the future, these approaches may be critical to predict the efficacy of novel drug combinations targeting the common driver alterations in CIN genes or pathways. 

In the context of CIN-targeted therapies, several beneficial combinatorial strategies can be envisioned. First, the anticipated drug resistance associated with a given targeted agent may be prevented through the combined effect of a second drug, often acting within the same pathway to inhibit that resistance mechanism [141]. For example, acquired BRAF inhibitor resistance in melanoma that results from the downstream activation of MEK (MAPK/ERK Kinase) effector proteins has been mitigated through combinatorial approaches targeting both BRAF and MEK [144,145]. It should be noted that *BRAF* is not an established CIN gene, however, the principle of exploiting resistance mechanisms against targeted agents could equally be applied in a CIN context when specific resistance mechanisms become more clearly understood. However, this approach does not eliminate the possibility for subsequent alterations in additional pathway members or compensatory pathways to impart drug resistance. Indeed, resistance to BRAF/MEK inhibition has been observed in up to one-third of melanoma patients, with genetic analyses revealing most had acquired either activating mutations in additional MAPK pathway members or in compensatory signaling pathways [146,147]. Thus, while combinatorial treatment approaches may prolong patient survival relative to single agents, drug resistance may still be a concern, particularly in the context of tumors with CIN.

An additional combinatorial approach that may benefit patients involves the simultaneous targeting of multiple CIN pathways. Conceivably, this approach may better address the multiple mechanisms giving rise to CIN and ITH in a given tumor or patient. For example, combining a microtubule-stabilizing drug (e.g., Paclitaxel) with a SAC inhibitor is still expected to induce death, should taxane resistance arise. In fact, preclinical work evaluating Paclitaxel and inhibitors of the SAC component MPS1 (Monopolar Spindle 1) has revealed a synergistic interaction suggesting that the disruption of one CIN pathway may potentiate the disruption of a second CIN pathway [148,149,150]. In further support of this possibility, a synergistic interaction has also been observed between Aurora kinase A and MPS1 inhibitors [151]. Importantly, synergistic drug combinations may permit dose reductions of either or both therapeutic agents, that may minimize the off-target side effects associated with each drug [152]. Furthermore, it has been suggested that drug combinations that target distinct cellular processes are less likely to exhibit additive toxicities in healthy cells associated with combinations targeting a single pathway [153].

A final combinatorial strategy seeks to merge a CIN-exploiting compound with a conventional chemotherapy that exhibits more a generalized cytotoxic effect, like DNA damaging agents. While resistance to targeted therapies frequently occurs due to mutations in the target gene or by compensatory pathway activation, there may be fewer possible mechanisms of resistance associated with non-specific cytotoxic agents that act randomly on highly conserved structures, such as DNA. For example, while genetic adaptation to CIN is a possible mechanism contributing to Paclitaxel resistance, these cells are expected to remain sensitive to Carboplatin, a conventional chemotherapy and DNA alkylating/cross-linking agent that prevents DNA replication and induces cell death [154]. For the past two decades, combinations of Paclitaxel with various platinum agents, including Cisplatin and Carboplatin, have been the standard of care in advanced epithelial ovarian cancer [155,156,157]. Thus, through strategic design, combinatorial treatment approaches have the potential to induce robust killing. In addition, the many possible pair-wise combinations of CIN-exploiting drugs with additional chemotherapeutic agents vastly increase the overall number of therapeutic options. However, identifying effective and clinically relevant drug combinations requires careful consideration, to achieve a precise balance between optimal efficacy and tolerability.

## 9. Future Directions and Considerations for Targeting and Exploiting CIN in Cancer

In the future, precision medicine strategies that exploit CIN may become effective treatment options for both primary and metastatic disease. However, before this can be realized, a more comprehensive understanding of the aberrant genes and mechanisms giving rise to CIN is necessary. To identify multiple actionable targets for optimal combination-based therapy, an in-depth analysis of a patient’s tumor(s) will be required. Unfortunately, this therapeutic strategy is not currently feasible, as we lack a comprehensive list of genes and biological pathways that underlie CIN. In addition, the methods employed to detect and assess CIN in patient samples are costly, time-consuming, technically challenging, and are not readily available for routine clinical use. Nevertheless, as new screening approaches are developed to rapidly assess CIN, and as the costs of DNA sequencing continue to decrease, the current targeted therapeutic arsenal that exploits CIN is likely to become a highly effective strategy in the fight against cancer.

CIN-exploiting treatment strategies will continue to evolve as we gain a greater fundamental understanding of this aberrant phenotype. Interestingly, recent work by Shaukat and colleagues [158] demonstrated that numerical CIN sensitizes cells to oxidative stress arising from metabolic alterations. More specifically, they showed that targeted knock-down of metabolic genes in CIN-positive cells induced increases in oxidative damage, DNA double-strand breaks and apoptosis, resulting in selective killing of cancer cells with CIN. Additional studies have shown that increases in reactive oxygen species following SOD1 (Superoxide Dismutase 1) silencing and inhibition induces SL killing in colorectal cancer cells harboring defects in established CIN genes, like *RAD54B*, *BLM*, and *CHEK2* [129,140]. Although early, these results suggest that metabolic reprogramming, a hallmark of cancer cells [7], may represent a future broad-spectrum therapeutic target for cancers that exhibit CIN.

## 10. Conclusions

CIN-exploiting therapies represent an innovative strategy with great therapeutic potential and broad-spectrum applicability for cancer patients. As we gain greater insight into the altered genes and aberrant pathways driving CIN, our ability to identify and develop novel therapeutics that exploit those aberrant origins will greatly expand. Fundamental research aimed at identifying the molecular origins of CIN and determining the impact on tumor development, evolution, and metastasis are critical to address many unanswered questions, including: (1) What are the aberrant genes and biological processes that give rise to CIN and contribute to tumor development, metastasis, and the acquisition of drug resistance? (2) What factors determine a cell’s ability to survive with CIN and are these cell-, tissue- or tumor-specific? (3) Is there a critical CIN threshold that distinguishes tumor viability from lethality that can be exploited for therapeutic purposes? Answers to these questions, coupled with the continued advancement and clinical application of novel approaches to detect and assess CIN will be critical to effectuate appropriate precision medicine strategies in the future. Furthermore, as combinatorial therapeutic strategies continue to evolve, it is anticipated that synergistic combinations will be identified that are associated with minimal side effects, drug resistance, tumor recurrence or secondary cancers. Thus, studies evaluating the efficacy of new drug combinations that exploit CIN and SL interactors of CIN genes will be essential to direct and expand future treatment options. Accordingly, exploring and exploiting CIN has tremendous therapeutic potential in cancer, and may represent a critical vulnerability that can be targeted to treat aggressive, drug resistant cancers, to ultimately improve the quality of life and outcomes for those living with cancer.

## Figures and Tables

**Table 1:** Approaches to Assess CIN in vitro and in Patient Samples.

	Principle	Advantage	Limitation
Indirect Indicators of Chromosome Instability (CIN)			
Gene expression signatures	The genes of which the expression is most highly correlated with high levels of aneuploidy were selected to define the CIN70 expression signatures [31]. The signature was confirmed to correlate with structural and numerical CIN [37].	Applicable to published gene expression datasets for research purposes. Could be used in routine clinical practice with fresh or fixed patient samples.	No direct measurement of the level of CIN.
Array comparative genome hybridization (CGH)	The genome to be tested and a reference genome are labeled with distinct fluorescent probes and competitively hybridized to arrayed DNA sequences. The fluorescent signal indicates whether the test genome harbors a gain or loss of material at a given locus.	Detailed resolution of the recurrent copy number alterations present in the tumor.	No direct measurement of the level of CIN. Cannot distinguish between CIN and stable aneuploidy.
Nuclear area heterogeneity	Nuclear area is correlated with DNA content. Nuclear area heterogeneity is suggestive of underlying DNA content differences [38,39].	Observable in routine pathology reports.	Follow-up assessment with single-cell method is necessary to confirm nuclear area heterogeneity is due to CIN.
Micronucleus formation	Micronuclei contain missegregated chromosomes or large chromosomal fragments. An increase in micronucleus formation is indicative of DNA content changes/structural DNA damage [34,38,39].	Observable in routine pathology reports.	Follow-up assessment with single-cell method is necessary to confirm increased micronucleus formation is due to CIN.
Direct Measurements of CIN			
DNA image cytometry	Nuclei are extracted from the test samples and DNA is stained with the Feulgen method. Nuclei are microscopically imaged and optical density is recorded for each nucleus to calculate DNA content. Cell-to-cell variability is assessed with the Stemline Scatter Index (SSI), which is equal to the sum of the percentage of cells in the S-phase region, the percentage of cell with DNA content exceeding G2 and the coefficient of variation of the DNA content stemline [40,41].	Higher throughput than FISH. Applicable to formalin fixed, parafin embedded (FFPE) tumor samples.	No distinction between numerical and structural CIN.
Fluorescence in situ Hybridization (FISH)	Fluorescent probes detect centromere copy numbers to assess gain or loss of chromosomes in individual cells [8,10,42,43]. Alternatively, probes binding to chromosomal arms can be employed to assess segmental aneuploidy and structural CIN [34].	Accurate measurement of cell-to-cell heterogeneity. Hundreds of cells evaluated at a time. Applicable to FFPE tumor samples.	Labor intensive. Microscope capacity generally limits the analysis to 3–4 probes at a time. Cannot measure structural and numerical CIN at the same time.
Single-cell CGH	CGH is performed to analyze the DNA of individual tumor cells after amplification of their genome. CIN level in the tumor samples is inferred from degree of cell-to-cell heterogeneity.	High resolution of copy number alterations present in individual cells.	Lower resolution of copy number alterations than single-cell sequencing. Technology still under development, not yet reliably applicable in clinical setting.
Single-cell sequencing	New generation sequencing technology is applied to isolated single tumor cells after amplification of their genome. Copy number variations can be assessed across the whole genome. CIN level is inferred from degree of cell-to-cell heterogeneity.	Detailed resolution of copy number alterations Base-pair resolution of mutations.	Technology still under development, not yet reliably applicable in clinical setting.

**Table 2:** Classes and Mechanisms of Numerical CIN-Inducing and -Reducing Therapies.

Drug Family	Mechanism of Action	Effect on CIN	Drug Examples	Molecular Target	Clinical Trials	Clinicaltrials.gov Identifier [60]
Microtubule Dynamics						
Microtubule stabilizers (taxanes, epothilones)	Bind tubulin subunits to inhibit microtubule depolymerization	I [54,61]	Paclitaxel	β-tubulin	FDA approved (breast, ovarian, non-small cell lung cancer, Kaposi sarcoma)	FDA approved
			Docetaxel	β-tubulin	FDA approved (breast, prostate, gastric, head & neck, non-small cell lung cancer)	FDA approved
			Ixabepilone	β-tubulin	FDA approved (breast)	FDA approved
Microtubule destabilizers (vinca alkaloids, colchicine analogs)	Inhibit microtubule polymerization and induce mitotic arrest	U	Vincristine	β-tubulin	FDA approved (leukemia)	FDA approved
			Vinblastine	β-tubulin	FDA approved (breast, testicular, Hodgkin lymphoma, non-Hodgkin lymphoma, Kaposi sarcoma)	FDA approved
			Vinorelbine	β-tubulin	FDA approved (non-small cell lung cancer)	FDA approved
Mitotic Checkpoints						
Spindle assembly checkpoint inhibitors	Induce premature mitotic exit and chromosome missegregation	I [62,63]	BAY1217389	MPS1 [64]	Phase I	NCT02366949
			BAY1161909	MPS1 [64]	Phase I	NCT02138812
			CFI-402257	MPS1 [65]	Phase I	NCT02792465
Anaphase- promoting complex/cyclosome (APC/C) inhibitors	Inhibit mitotic exit and induce metaphase arrest	R [66]	Tosyl-L-arginine methyl ester (TAME)	APC/C [67]	Preclinical [68,69]	Preclinical
Mitotic Kinases						
Aurora kinase inhibitors	Interfere with mitotic chromosome alignment, spindle assembly, and cytokinesis	I [70,71]	ENMD-2076	Aurora Kinase A [72,73]	Phase II	NCT01104675 NCT01639248
			Alisertib (MLN8237)	Aurora Kinase A [74]	Phase I/II	NCT02187991 NCT01923337
			Barasertib (AZD1152)	Aurora Kinase B [74]	Phase II/III	NCT00952588
			GSK1070916	Aurora Kinase B/C [75]	Phase I	NCT01118611
Polo-like kinase inhibitors	Inhibit bipolar spindle formation, sister chromatid separation, and cytokinesis	I [76,77,78]	Volasertib (BI 6727)	PLK1 [78]	Phase I/II	NCT02273388 NCT01121406
			BI 2536	PLK1 [78]	Phase II	NCT00706498 NCT00710710
			Rigosertib (ON 01910.Na)	PLK1 [78]	Phase I/II	NCT01168011 NCT01807546
Microtubule-associated Motor Proteins						
KIF11 (Eg5) inhibitors	Interfere with centrosome separation and cause monopolar spindle formation	I [19] or R [79]	Filanesib (ARRY-520)	KIF11 [80]	Phase I/II	NCT00821249
			MK0731	KIF11 [81]	Phase I	NCT00104364
KIFC1 (HSET) inhibitors	Inhibit centrosomal clustering activity of KIFC1, resulting in multipolar spindle formation	I [82]	CW069	KIFC1 [82]	Preclinical [83,84]	Preclinical
			PJ34	KIFC1 [82]	Preclinical [85]	Preclinical
CENP-E inhibitors	Inhibit CENP-E mediated chromosomal alignment in metaphase	I [86]	GSK923295	CENP-E [86]	Phase I	NCT00504790
			PF-2771	CENP-E [87]	Preclinical	Preclinical
KIF2C potentiators	Enhance KIF2C activity and destabilize kinetochore-microtubule attachments, leading to a reduction in erroneous attachments	R [52]	UMK57	Uncharacterized	Preclinical	Preclinical
Centrosome Dynamics						
Centrosomal clustering inhibitors	Inhibit supernumerary centrosomal clustering, leading to multipolar spindle formation	I [88]	CCCI01	Uncharacterized	Preclinical [88]	Preclinical
Chromatin Modification						
Histone deacetylase (HDAC) inhibitors	Accumulation of acetylated histones disrupts centromere function and causes mitotic abnormalities	I [89,90]	Romidepsin	HDAC1/2 [91]	FDA approved (cutaneous T-cell lymphoma)	FDA approved
			Entinostat	Class I HDACs [92]	Phase I/II	NCT01105377 NCT00020579
			Vorinostat	Class I/II HDACs [93]	Phase I/II	NCT01045538 NCT00365599

^1^ Effect on CIN characterized as: I = Inducing, R = Reducing, or U = Uncharacterized. FDA: The Food and Drug Administration; MPS1: Monopolar Spindle 1; PLK1: Polo Like Kinase 1; KIF11: Kinesin Family Member 11; KIFC1 (HSET): Kinesin Family Member C1; KIF2C; Kinesin Family Member 2C; CENP-E: Centromere Protein E.
